# Evaluation and Characterization of Post-Stroke Lung Damage in a Murine Model of Cerebral Ischemia

**DOI:** 10.3390/ijms23158093

**Published:** 2022-07-22

**Authors:** Júlia Faura, Laura Ramiro, Alba Simats, Feifei Ma, Anna Penalba, Teresa Gasull, Anna Rosell, Joan Montaner, Alejandro Bustamante

**Affiliations:** 1 Neurovascular Research Laboratory, Valld’Hebron Research Institute (VHIR), Universitat Autònoma de Barcelona (UAB), 08035 Barcelona, Spain; juliafaura10@gmail.com (J.F.); laura.ramirop@gmail.com (L.R.); simatsalba@gmail.com (A.S.); mafeifeivhir@gmail.com (F.M.); anna.penalba@vhir.org (A.P.); anna.rosell@vhir.org (A.R.); jmontaner-ibis@us.es (J.M.); 2 Cellular and Molecular Neurobiology Research Group, Department of Neurosciences, Germans Trias i Pujol Research Institute (IGTP), 08916 Badalona, Spain; tgasull@igtp.cat; 3 Stroke Research Program, Institute of Biomedicine of Seville, IBiS/Hospital Universitario Virgen del Rocío/CSIC, University of Seville, 41013 Seville, Spain; 4 Department of Neurology, Hospital Universitario Virgen Macarena, 41009 Seville, Spain; 5 Stroke Unit, Hospital Universitari Germans Trias i Pujol, 08916 Badalona, Spain

**Keywords:** stroke, inflammation, pulmonary complications

## Abstract

After stroke and other brain injuries, there is a high incidence of respiratory complications such as pneumonia or acute lung injury. The molecular mechanisms that drive the brain-lung interaction post-stroke have not yet been elucidated. We performed transient middle cerebral artery occlusion (MCAO) and sham surgery on C57BL/6J mice and collected bronchoalveolar lavage fluid (BALF), serum, brain, and lung homogenate samples 24 h after surgery. A 92 proteins-panel developed by Olink Proteomics^®^ was used to analyze the content in BALF and lung homogenates. MCAO animals had higher protein concentration levels in BALF than *sham*-controls, but these levels did not correlate with the infarct volume. No alteration in alveolar-capillary barrier permeability was observed. A total of 12 and 14 proteins were differentially expressed between the groups (FDR < 0.1) in BALF and lung tissue homogenates, respectively. Of those, HGF, TGF-α, and CCL2 were identified as the most relevant to this study. Their protein expression patterns were verified by ELISA. This study confirmed that post-stroke lung damage was not associated with increased lung permeability or cerebral ischemia severity. Furthermore, the dysregulation of HGF, TGF-α, and CCL2 in BALF and lung tissue after ischemia could play an important role in the molecular mechanisms underlying stroke-induced lung damage.

## 1. Introduction

After acute brain injuries such as stroke, traumatic brain injury (TBI), and subarachnoid hemorrhage, there is a high incidence of lung-related complications, such as pneumonia, acute lung injury (ALI), acute respiratory distress syndrome (ARDS), and neurogenic pulmonary edema [1,2]. The high frequency of lung damage after a brain injury suggests a very close interaction between these organs after the episode. This interaction seems bidirectional, though the exact mechanisms are not yet fully elucidated. After brain injury, proinflammatory cytokines are secreted in the brain, the brain-blood barrier (BBB) is impaired, facilitating leukocyte infiltration into the brain. These events involve several systemic alterations that can damage the peripheral organs, including the lungs. This local peripheral damage, in turn, exacerbates the primary damage in the brain. This bidirectional relationship between the brain and the lungs has been termed the “double hit model” [3].

The local effect of stroke on the lungs has become the focus of recent research efforts. In an experimental rat model of cerebral ischemia, both pulmonary edema and ultrastructural changes in the lung parenchyma were observed after ischemia. In addition, levels of proinflammatory cytokines, including interleukin-6 (IL-6) and tumor necrosis factor-α (TNF-α), were elevated in the plasma, brain, and bronchoalveolar lavage fluid (BALF) [4]. In contrast, Austin et al. [5] did not observe lung damage (i.e., edema or impaired lung function) after cerebral ischemia in mice. However, they did observe inflammation in this organ through the expression of pro-inflammatory cytokines in both lung tissue and BALF. Also, the immune niche of the lung is dysregulated after cerebral ischemia, as characterized by a decrease in lymphocytes and an increase in alveolar macrophages and neutrophils [6].

Stroke-associated pneumonia (SAP) is one of the most frequent complications after a stroke, with an incidence of around 12% [7], and increases the risk of mortality and disability in stroke patients and their clinical length-of-stay [8]. Nowadays, there is a lack of preventive therapies for SAP in clinical practice. Clinical trials testing antibiotic prophylaxis have not succeeded either in reducing the SAP incidence or improving the prognosis of stroke patients [9]. As stroke-induced immunosuppression is one of the major factors for the occurrence of SAP, immunomodulating therapies have also been proposed for SAP prevention, with great success in experimental studies but still moderate translation into human-based studies [10]. Hence, identifying novel therapeutic targets and therapies for SAP prevention is urgently needed in this field of study.

Therefore, this study aims to elucidate the mechanisms that can drive stroke-induced lung damage by characterizing BALF and lung protein content to identify potential therapeutic targets for the prevention of SAP.

## 2. Results

### 2.1. BALF Characterization after Cerebral Ischemia

**Figure 1:**
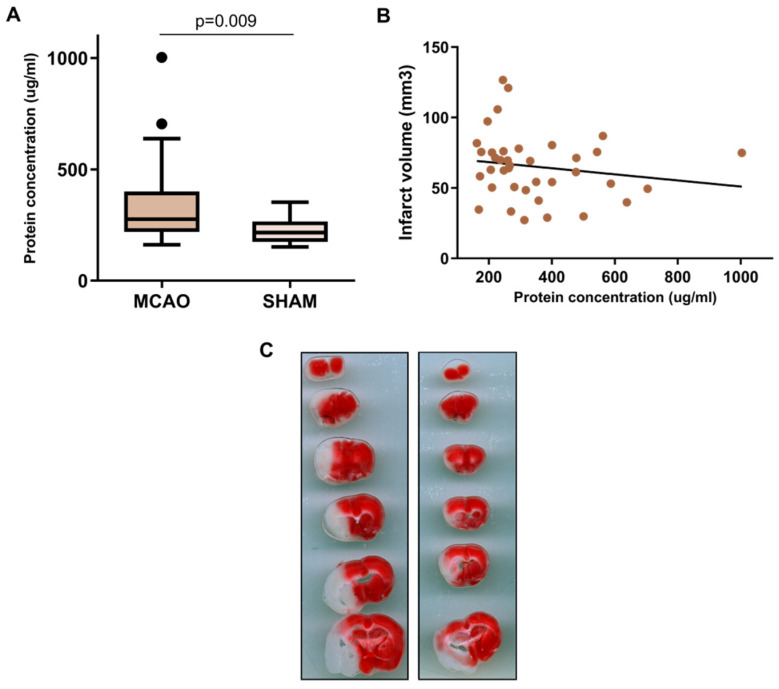
Evaluation of the protein concentration in BALF. (**A**) Boxplot comparing the BALF protein concentration between ischemic and sham mice (N = 53) (**B**) Scatter plot representing the BALF protein concentration (*X* axis) and the infarct volume (*Y* axis) of the ischemic animals (N = 42). (**C**) Two representative images of cerebral ischemia measured by TTC staining. The boxplot in (**A**) shows increased protein concentration in MCAO animals compared to *sham*, while the scatterplot in B showed the lack of correlation between infarct volume and protein concentration. MCAO: middle cerebral artery occlusion.

To characterize the impact of stroke on the lungs, we evaluated the BALF protein content in 42 ischemic and 11 *sham-control* mice. The total protein concentration increased in ischemic compared with *sham*-control animals (275.7 (interquartile range (IQR): 218.4–400.6) μg/mL vs. 215.8 (174.6–266.1) μg/mL), *p* = 0.009, N = 42 vs. 11) (Figure 1A). No correlation was observed between BALF protein content and the severity of the ischemia, as evaluated by the infarct volume (in mm^3^) (Figure 1B).

**Figure 2:**
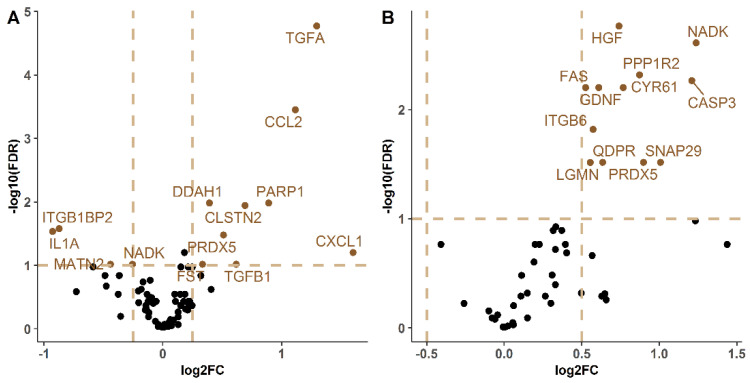
Volcano plot of significant proteins in the BALF (**A**) and lung (**B**) analyses of ischemic vs. *sham* animals. The *X*-axis represents the logFC and the Y-represents the logarithm in base 10 of the False Discovery Ratio (FDR). The horizontal line symbolizes an FDR < 0.1 and the vertical lines symbolize an FDR > |0.25|. Proteins over or under the mentioned cut-off points for FDR and FC represent the differentially expressed proteins and are marked in brown. A total of 12 proteins (BALF) and 14 (lung) showed differential expression between ischemic and *sham* animals. LogFC: logarithmic fold-change, FDR: false discovery ratio.

Next, we aimed to characterize the protein content in BALF to identify molecular alterations caused by cerebral ischemia. For this, we analyzed protein content by simultaneously measuring 92 molecules in the BALF samples from 42 ischemic and 11 *sham-control* animals. Proteins with more than 40% of their values below the detection limit were excluded, and of the 49 remaining, 12 were differentially expressed (FDR < 0.1) between the two groups. All of these differentially expressed proteins were upregulated in the ischemic animals. The most significant proteins were hepatocyte growth factor (HGF), NAD kinase (NADK), and protein phosphatase inhibitor 2 (PPP1R2) (Table 1, Figure 2A).

**Figure 3:**
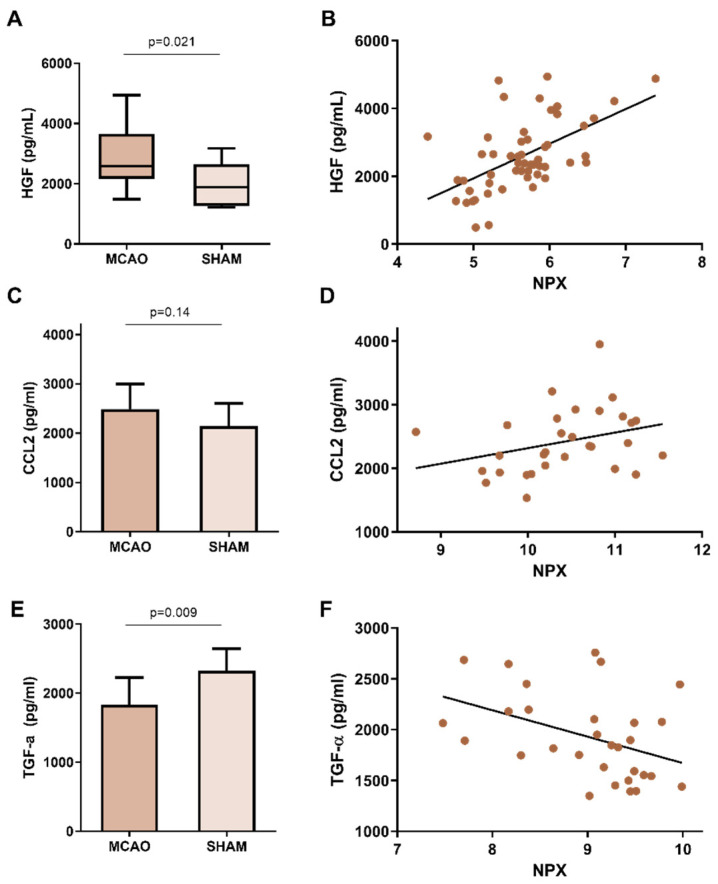
HGF, CCL2 and TGF-α measurements by ELISA. (**A**) Boxplot comparing HGF levels in BALF of MCAO vs. *sham* mice (N = 49) (**B**) Dotplot representing HGF measurement by Olink^®^ panel (*X*-axis) and measurement by ELISA (*Y* axis) (N = 53). (**C**) Boxplot comparing CCL2 levels in lung of MCAO vs. *sham* mice (N = 30) (**D**) Dot plot representing CCL2 measurement by Olink^®^ panel (*X* axis) and ELISA measurement (*Y* axis) (N = 30). (**E**) Boxplot comparing TGF-α levels in lung of MCAO vs. sham mice (N = 30) (**F**) Dot plot representing TGF-α measurement by the Olink^®^ panel (*X* axis) and ELISA measurement (*Y* axis) (N = 30). The boxplots showed increased HGF, a trend towards increased CCL2 and decreased TGF-a values in MCAO vs. *sham* animals, while the scatterplots showed positive correlations for HGF and CCL2 and a negative correlation for TGF-a. HGF: Hepatocyte Growth Factor, CCL2: C-C Motif Chemokine Ligand 2, TGF-α: Transforming Growth Factor α, ELISA: Enzyme-Linked ImmunoSorbent Assay, BALF: bronchoalveolar lavage fluid, NPX: normalized protein expression, MCAO: middle cerebral artery occlusion.

An increase in the HGF levels in the BALF of the ischemic animals compared with *sham* animals was confirmed using another technique (ELISA) (2541.3 (2051.35–3491.26) pg/mL vs. 1887.31 (1283.22–2496.97) pg/mL, *p* = 0.021) (Figure 3A). HGF levels measured by both techniques (Proximity Extension Assay (PEA) and Enzyme-Linked ImmunoSorbent Assay (ELISA) positively correlated (R = 0.56, *p* < 0.0001) (Figure 3B).

### 2.2. Protein Expression Characterization in Lung

Next, we aimed to characterize the protein content in whole lung homogenates to identify new molecules deregulated in the lung after stroke. To that end, the same 92 molecules were simultaneously measured in the lung homogenates from the same 24 ischemic and 6 *sham-control* animals.

Out of the 75 proteins included in the final analysis after filtering by expression, 14 differentially expressed between experimental conditions were found. Four of these were downregulated in MCAO mice, while the remaining ten were upregulated after stroke. Among the significantly dysregulated proteins, only two showed significant differences between the two groups in BALF and lung homogenates. These were NADK and mitochondrial peroxiredoxin-5 (PRDX5). NADK was upregulated in the BALF of ischemic mice and downregulated in the lung, while PRDX5 was upregulated in both samples from ischemic mice.

In this case, Transforming Growth Factor-α (TGF-α) in its proform (proTGF-α), C-C Motif Chemokine Ligand 2 (CCL2), and dimethylarginine dimethylaminohydrolase 1 (DDH1) were identified as the most significant proteins of this analysis. Table 1 shows the logFCs and *p*-values of the differentially-expressed proteins plotted in Figure 2B.

Next, the TGF-α and CCL2 findings were further verified by an alternative technique (ELISA) in the same lung homogenate samples. A slight increase in CCL2 was confirmed by ELISA in ischemic compared with *sham* animals. However, this difference did not reach statistical significance (2487 (±508.4) pg/mL vs. 2145 (±459.5) pg/mL, *p* = 0.14) (Figure 3C). A positive correlation was also observed between the ELISA and Olink^®^ values, with a correlation coefficient of 0.66 (*p* < 0.0001) (Figure 3D).

Regarding TGF-α, the ELISA corroborated that lower levels were present in the lung homogenates from ischemic compared with *sham* mice (1833 (±393.2) pg/mL vs. 2323 (±322.8) pg/mL, *p* = 0.009) (Figure 3E), which negatively correlated with the values obtained by Olink^®^ (R = −0.42, *p* = 0.02) (Figure 3F). However, the Olink^®^ panel detects proTGF-α, while commercial ELISAs measure the mature form of TGF-α.

### 2.3. Alveolar-Capillary Barrier Permeability

**Figure 4:**
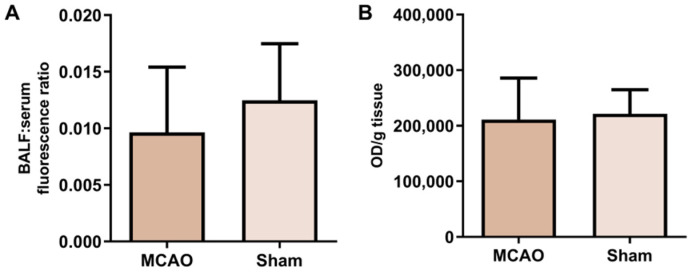
Lung permeability assessment. (**A**) Bar plot representing the ratio between BALF and serum fluorescence (N = 7 vs. 5). (**B**) Bar plot representing the amount of fluorescence of the tissue, adjusted by the weight of each organ (N = 7 vs. 5). For both comparisons, no significant differences were noted between MCAO and *sham* animals. BALF, bronchoalveolar lavage fluid; OD, optical density.

We next examined whether the increase in BALF protein concentration was caused by an increase in epithelial permeability in the lungs of ischemic animals. FITC-dextran was injected into 7 animals undergoing MCAO and 5 *sham*-control animals, and the ratio of BALF to serum fluorescence was determined. No differences were observed between the ratios of the two groups (Figure 4A). In addition, the amount of FITC-dextran that penetrated the lung was also studied. In this case, no differences were found between the animals subjected to MCAO and the *sham* animals after adjusting the fluorescence of the lung by the weight of each tissue sample (Figure 4B).

## 3. Discussion

This study confirms that lung damage occurs after cerebral ischemia. According to our results, this damage was not associated with the impaired permeability of the alveolo-capillary barrier nor conditioned by the severity of brain ischemia. In addition, we identified and verified HGF, TGF-α, and CCL2 as dysregulated proteins in the lungs after cerebral ischemia.

Respiratory infections complicate the prognosis of patients, not only in stroke but also in other acute diseases of the central nervous system such as TBI. Pneumonia is also one of the most common complications in patients with severe TBI, with an incidence of about 30% [11]. Immunosuppression is a common feature of these two diseases, as is overcoming lung complications such as pneumonia, ALI, and ARDS, which highlights the crosstalk between the brain and lungs.

There are currently no preventive therapies for SAP available in clinical practice. Prophylactic antibiotics have been studied in multiple clinical trials, all of which indicate that these do not reduce SAP or mortality in stroke patients [9]. Immunomodulatory therapies represent a viable alternative. However, given that these treatments’ success is limited to experimental studies, additional research in this field is crucial [10]. Thus, understanding the local effects of stroke on the lung and its role in the susceptibility of stroke patients to SAP is a crucial means for identifying novel therapeutic targets.

Although the interaction between the brain and the lungs is well known, the effect of cerebral ischemia on the lung has not been studied until recently. We observed an increase in BALF protein concentration 24 h after ischemia in animals undergoing cerebral ischemia compared with sham animals, which indicates lung damage. These results align with Samary et al. [4], who observed an increase in total BALF protein concentration in rats 24 h post-focal ischemia and impaired ventilatory parameters such as tidal volume and respiratory rate.

While Austin et al. [5] did not find any differences in BALF protein concentration between MCAO and *sham* animals, they observed an increase in the total number of BALF cells, as well as macrophages and neutrophils counts. The sample size included in their study was smaller than the analysis presented here (N = 22 vs. 53), which may underlie this discrepancy.

We hypothesized that the permeability of the alveolar-capillary barrier was malleable and would increase after cerebral ischemia, similar to what has been described for the intestinal barrier [12]. The alveolar epithelium is a physical barrier primarily responsible for maintaining homeostasis within the lung that also protects this organ from external agents. This barrier is known to be altered in lung diseases such as ARDS, ALI, and COPD [13,14]. In the present study, we found no evidence of impaired permeability across the alveolar epithelium in ischemic mice at 4000 KDa. These results could be further supported by analyzing the expression of tight junction-related proteins such as the claudin family or occludin.

We also identified several proteins in BALF and lung homogenate whose expression levels were altered in ischemic mice relative to non-ischemic controls; three of these were selected for further investigation (HGF, CCL2, and TGF-α). HGF is a growth factor produced by mesenchymal cells (e.g., fibroblasts, macrophages) that activates signal transduction pathways involved in a diversity of biological processes, including cell migration, proliferation, and morphogenesis. The role of HGF in lung repair has been extensively characterized, and its upregulation in the BALF of ischemic mice has already been described in the literature. Moreover, HGF levels are increased in the BALF of patients with lung damage, specifically ARDS [15]. Similarly, an increase in HGF gene expression occurs after ALI induction in rats as a compensatory mechanism to mitigate the effects of the damage at a cellular level [16]. Our findings align with these previous studies, which further supports HGF as a reliable indicator of lung damage after cerebral ischemia.

In addition to HGF, the cytokine CCL2 was present at higher levels in the lung homogenates of MCAO mice compared with sham mice. CCL2 is involved in numerous pro-inflammatory processes, including in the lung, as reported by van Zoelen et al. [17]. Farris et al. [6] characterized the molecular changes to the immune niche in the lung after cerebral ischemia in mice and found CCL2 increased 24 h after ischemia relative to *sham* animals in lung homogenates. In that study, however, the difference in CCL2 levels disappeared within 72 h after ischemia.

Finally, we also studied the expression of TGF-α in the lung. The precursor form of this molecule, proTGF-α, is cut at the N-terminal and C-terminal sites to become the mature form [18]. Using the PEA technique, proTGF-α was detected in the samples, and higher levels of the precursor were observed in ischemic than *sham*-control animals. In contrast, for the validation of PEA technique, we used an ELISA that detects the mature form. The ELISA result was the opposite, with *sham* mice having the highest levels of this protein in its mature form. In addition, the correlation between the two techniques was negative, indicating an inversely proportional relationship. These results suggest that ischemia may influence the TGF-α maturation process. This molecule is a ligand of the epidermal growth factor receptor (EGFR) and activates signaling pathways in cell proliferation and differentiation, among others. In the lung, the expression of the gene encoding for TGF-α (TGFA) and EGFR increases after bleomycin-induced lung injury in rats, as does its protein expression four days after the insult [19]. Later, Hardie et al. induced lung injury in transgenic mice expressing TGF-α at different levels and found that animals with the highest expression levels exhibited an attenuated inflammatory response and reduced pulmonary edema, thus suggesting that this protein plays a protective role in lung damage [20,21].

The clinical implications of the lung damage associated with cerebral ischemia have yet to be fully determined. In addition to the damage mentioned above, an alteration in the lung immune niche increased the populations of alveolar macrophages and the infiltration of neutrophils into the tissue, and decreased lymphocyte counts. In addition, the expression of various cytokines (e.g., CCL5 and CCL22 decrease in the lung [6]). These results highlight stroke-associated lung damage as a process that contributes to the susceptibility of stroke patients to SAP. How these processes contribute remains unknown. We suggest that the lung damage could suppress local defenses in the alveoli, thus contributing to SAP susceptibility and that the ensuing protective, counteractive mechanisms maybe mediated by HGF and TGF-α, among others.

This study has some limitations. As discussed above, only one indicator was used to assess lung damage. In addition, the lungs of these animals were not histologically analyzed due to difficulties in processing lung samples. The small quantity of fluid obtained via BAL limited the number of candidates evaluated in the validation phase. However, the protein alterations were validated by two independent techniques using the same samples. External validation in another group of ischemic and *sham* mice would further support our results.

In conclusion, the present study confirmed that lung damage occurred after cerebral ischemia in mice. However, we demonstrated for the first time that this damage, in the present animal model, was not related to the increased permeability of the alveolar-capillary barrier or the severity of the ischemia. In addition, we identified three signaling molecules (HGF, TGF-α, and CCL2) involved in this specific form of lung damage, which might constitute key mediators of this damage. Our results provide deeper mechanistic insight into this unique pathological process, and we hope it will help to develop novel therapeutic approaches in SAP.

## 4. Materials and Methods

### 4.1. Experimental Design

All surgical procedures were carried out in compliance with Spanish legislation and according to the Directives of the European Union. This study was approved by the Ethics Committee of the Valld’Hebron Research Institute (protocol 03/19). All experiments were conducted in a randomized manner and adhered to the ARRIVE guidelines [22].

Male 8–12-week-old C57BL/6J mice were housed in a climate-controlled environment on a 12-h light/12-h dark cycle. Food and water were available ad libitum. Analgesia (buprenorphine, 0.05 mg/kg, s.c, DivasaFarma-Vic S.A, Barcelona, Spain) was administered to all animals to minimize pain and discomfort subcutaneously. Anesthesia (isoflurane, 4% for induction, 2% for maintenance in medical air oxygen, Abbot Laboratories, Barcelona, Spain) was delivered via facemask during all surgical procedures described below.

In 53 animals (42 ischemic and 11 *sham*-control), lung damage and BALF protein expression were assessed. In addition, in 30 of these 53 animals, protein expression was profiled from lung homogenates (24 ischemic and 6 *sham*-control). A different set of 12 animals (7 ischemic and 5 *sham*-control) were used for the lung permeability assay. Therefore, a total of 65 animals were used in the present study. The sample size was chosen according to sample and animal availability.

### 4.2. MCAO Surgery

Transient ischemia in the middle cerebral artery (MCA) was induced by introducing an intraluminal filament through the external carotid artery, as previously described [23]. Briefly, the animals were anesthetized, and their body temperature was maintained at 37 °C using a heating pad. The regional cerebral blood flow (CBF) was monitored near the region irrigated by the MCA during the whole process by affixing a laser Doppler probe (Moor Instruments, Axminster, UK) to the skull. Afterward, animals were placed in the supine position and the right bifurcation of the external carotid artery and internal carotid artery were exposed. Then, a silicone-coated nylon monofilament (Doccol Corporation, Sharon, MA, USA) was introduced through the external carotid artery to occlude the MCA. MCA occlusion (MCAO) was confirmed by a reduction in the cortical CBF recorded by the laser Doppler probe. Next, the incision was closed with a silk suture and animals were allowed to recover from the anesthesia. Ninety minutes later, the mice were re-anesthetized and the filament was removed to reperfuse the MCA. After reperfusion, the mice recovered for 24 h before being euthanized. Only animals with an 80% reduced CBF after filament introduction and an 80% recovery after filament removal were included in the study. *Sham*-control animals underwent the same surgical procedures without the insertion of the nylon filament, and therefore, without MCAO.

### 4.3. Sample Collection and Infarct Volume Quantification

Animals were anesthetized 24 h after surgery, and blood was collected by cardiac puncture. Lungs were washed five times by inserting a catheter through the trachea filled with an initial volume of 500 μL of saline solution and approximately 300 μL of BALF were collected after the last wash [24]. Next, the mice were trans-cardially perfused with 20 mL of cold saline solution and, immediately thereafter, the lungs and brains were quickly removed.

The brains were sectioned into six 1-mm slices under cold conditions and then stained with 2,3,5-triphenyltetrazolium chloride (TTC; Sigma-Aldrich, Madrid, Spain) to measure the infarct volume, as previously described [25]. TTC images were captured with a CanoScan 4200F scanner (Canon, Tokyo, Japan) and quantified with Image J software. Infarct volumes were calculated as previously described [26]. After the images were acquired, the slice corresponding to the bregma anatomical point (representing the core of the infarct tissue) was carefully dissected to separate the right (ipsilateral, IP) and left (contralateral, CL) hemispheres. Each hemisphere was flash-frozen in liquid nitrogen and stored at −80 °C.

Whole blood samples were centrifuged at 1500× *g*, 4 °C for 15 min. The serum was collected and stored at −80 °C. BALF samples were centrifuged at 12,000× *g*, 4 °C for 12 min, and the supernatant was collected and stored at −80 °C.

### 4.4. BALF Protein Concentration Quantification

The Bradford method was used to quantify the BALF protein concentration [27]. First, the following phosphatase and protease inhibitors were added to the samples: 1% PMSF (Sigma-Aldrich, Madrid, Spain), 0.5% Aprotinin (bovine lung-derived, Sigma-Aldrich), and 0.5% phosphatase inhibitor cocktail-3 (Sigma-Aldrich). Next, the Coomassie Protein Assay Reagent (ThermoFisher Scientific, Cornellà de Llobregat, Spain) was used for the quantification, according to the manufacturer’s instructions. Absorbance was measured at 595 nm with the Synergy™ Mx microplate reader (Biotek Instruments, Bad Friedrichshall, Germany). All samples were run in duplicate, and those with a coefficient of variation (CV) higher than 20% were re-run. An inter-plate control was used to assess the intra-assay variability.

### 4.5. Lung Permeability Assay

To quantify lung permeability, fluorescein isothiocyanate (FITC)-dextran, 4 kDa (Sigma-Aldrich) was intravenously injected via the retroorbital sinus at 20 mg/kg in a total volume of 100 μL.

Fluorescence was read in BALF and serum samples using the Synergy™ Mx microplate reader (Biotek Instruments) at 492 nm (emission) and 515 nm (detection). The amount of FITC-dextran retained in the lung was also measured. To that end, the right lungs were immersed in Formamide (ThermoFisher Scientific, Cornellà de Llobregat, Spain) at 37 °C. After 24 h, the samples were centrifuged at 15,000× *g* for 10 min, and the supernatants’ fluorescence was measured at the aforementioned wavelengths. Fluorescence values were normalized to the weight of each lung at the moment of the sacrifice.

### 4.6. Lung Homogenization

The left lungs of 24 ischemic and 6 *sham*-control mice were homogenized with fresh, cold lysis buffer (0.5% phosphatase inhibitor cocktail 3 (Sigma-Aldrich), 1% PMSF (Sigma-Aldrich), and 0.5% Aprotinin (Sigma-Aldrich) diluted in RIPA buffer (Sigma-Aldrich)). Lysis buffer (500 μL) was added to each sample, and the tissue was homogenized with a homogenizer drill (5 mm diameter). The samples were then centrifuged at 12,000 rpm, 4 °C for 12 min. The supernatants were stored at −80 °C and the pellets were discarded.

The total protein concentration of the lung homogenates was measured using the BCA (*bicinchoninic acid*) method [28] (Pierce^TM^ BCA Protein Assay, ThermoFisher), according to the manufacturer’s instructions. Absorbance was read at 562 nm with the Synergy™ Mx microplate reader (Biotek Instruments). All samples were run in duplicate, and those with a CV higher than 20% were re-run.

### 4.7. BALF and Lung Protein Characterization

The protein content of BALF and lung homogenates was characterized externally by Olink Proteomics^®^ (Uppsala, Sweden) using a proteomics assay that simultaneously measures 92 proteins per sample, as previously described [29]. In brief, this PEA involves 92 protein-specific antibody pairs labeled with unique complementary oligonucleotides (PEA probes) being added to 1 μL of sample in a 96-well-plate. Only when both antibodies in the pair bind to the corresponding protein, are their attached probes close enough to hybridize, which generates a polymerase chain reaction (PCR) target sequence that is subsequently amplified and detected using a standard real-time PCR protocol.

### 4.8. ELISA Protein Measurement

Three proteins were evaluated per sample using ELISA: HGF (Mouse HGF DuoSet ELISA, R&D Systems, TGF- α (Mouse TGF-α ELISA Kit, Elabscience, Bethesda, MD, USA), CCL2 (Mouse CCL2/JE/MCP-1 DuoSet ELISA, R&D Systems, Minneapolis, MN, USA). The assay procedures were performed according to the manufacturer’s instructions. Optical densities were determined with the Synergy™ Mx microplate reader (BioTek Instruments, Winooski, VT, USA). All samples were analyzed in duplicate and replicates with a CV greater than 20% were excluded from the statistical analyses.

### 4.9. Statistical Analyses

R software (version 3.6.1; R Foundation for Statistical Computing, Vienna, Austria) and Statistical Packages for Social Sciences (version 22; SPSS Inc., Armonk, NY, USA) were used for the statistical analyses and GraphPad Prism for generating plots (version 6; GraphPad Software, San Diego, CA, USA).

The distribution normality of the variables was analyzed graphically and with the Kolmogorov-Smirnov test if the sample size exceeded 30 and the Shapiro-Wilk’s test when the sample size was less than 30. For the univariate analyses, unpaired Student’s *t*-test was used to determine the statistical significance of normally distributed variables and the Mann-Whitney U test if the variables did not exhibit normal distribution. For the correlations, Spearman’s Rho or Pearson’s coefficient was used depending on the normality of the variables’ distribution. Normal variables are represented as means (±standard deviation (SD)) and non-normal variables are represented as medians (interquartile range (IQR)).

To analyze the data generated using the Olink Proteomics^®^ (Uppsala, Sweden) Mouse Exploratory Panel, proteins with more than 40% of their values below the detection limit were removed. Thereafter, differential expression analysis was carried out using linear Bayes models in the *limma* Bioconductor package [30]. For the multiple comparisons correction, the false discovery ratio (FDR) was applied, and logFC was used to analyze the magnitude of the differences. Statistical significance was established when the absolute FDR was higher than 0.1.

## Figures and Tables

**Table 1:** Top table of BALF (left) and lung homogenate (right) protein expression. Significant proteins are shown. The *p*-value column corresponds to the unadjusted *p*-value. logFC: logarithmic fold-change, FDR: false discovery ratio.

BALF					Lung Homogenate				
Protein	logFC	*p*-Value	FDR	Uniprot Code	Protein	logFC	*p*-Value	FDR	Uniprot Code
Hepatocyte growth factor (HGF)	0.740	>0.001	0.002	Q08048	Protransforming growth factor alpha (TGFA)	1.296	>0.001	>0.001	P48030
NAD kinase (NADK)	1.237	>0.001	0.002	P58058	C-C motif chemokine 2 (CCL2)	1.115	>0.001	>0.001	P10148
Protein phosphatase inhibitor 2 (PPP1R2)	0.873	>0.001	0.005	Q9DCL8	N(G),N(G)-dimethylarginine dimethylaminohydrolase 1 (DDAH1)	0.392	0.001	0.010	Q9CWS0
Caspase-3 (CASP3)	1.209	>0.001	0.005	P70677	Poly [ADP-ribose] polymerase 1 (PARP1)	0.891	0.001	0.010	P11103
CNN family member 1 (CYR61)	0.767	0.001	0.006	P18406	Calsyntenin-2 (CLSTN2)	0.691	0.001	0.011	Q9ER65
Glial cell line-derived neurotrophic factor (GDNF)	0.609	0.001	0.006	P48540	Integrin beta-1-binding protein 2 (ITGB1BP2)	−0.873	0.002	0.026	Q9R000
Tumor necrosis factor receptor superfamily member 6 (FAS)	0.524	0.001	0.006	P25446	Interleukin-1 alpha (IL1A)	−0.928	0.003	0.029	P01582
Integrin beta-6 (ITGB6)	0.573	0.002	0.015	Q9Z0T9	Peroxiredoxin-5 (PRDX5)	0.510	0.004	0.033	P99029
Peroxiredoxin-5 (PRDX5)	0.897	0.007	0.030	P99029	Disintegrin and metalloproteinase domain-containing protein 23 (ADAM23)	0.182	0.008	0.063	Q9R1V7
Synaptosomal-associated protein 29 (SNAP29)	1.007	0.007	0.030	Q9ERB0	Growth-regulated alpha protein (CXCL1)	1.600	0.008	0.063	P12850
Dihydropteridine reductase (QDPR)	0.634	0.007	0.030	Q8BVI4	Follistatin (FST)	0.333	0.015	0.096	P47931
Legumain (LGMN)	0.554	0.007	0.030	O89017	Matrilin-2 (MATN2)	−0.441	0.017	0.096	O08746
					NAD kinase (NADK)	−0.256	0.017	0.096	P58058
					Transforming growth factor beta-1 proprotein (TGFB1)	0.618	0.018	0.096	P04202

## Data Availability

The data presented in this study are available on request from the corresponding author.
